# Ultra-processed foods, dietary diversity and micronutrient intakes in the Australian population

**DOI:** 10.1007/s00394-023-03245-2

**Published:** 2023-10-05

**Authors:** Zeinab Houshialsadat, Gustavo Cediel, Isabela Sattamini, Gyorgy Scrinis, Priscila Machado

**Affiliations:** 1 School of Public Health, Physiotherapy and Sports Science, University College Dublin, Dublin, Ireland; 2 Ecole des hautes études en santé publique, Paris, France; 3 Escuela de Nutrición y Dietética, Universidad de Antioquia, Medellín, Colombia; 4 Department of Noncommunicable Diseases and Mental Health, Pan-American Health Organization/World Health Organization, Washington, DC USA; 5 School of Agriculture and Food, University of Melbourne, Melbourne, Australia; 6 Institute for Physical Activity and Nutrition, School of Exercise and Nutrition Sciences, Deakin University, Geelong, Australia

**Keywords:** Ultra-processed food, Micronutrient, Dietary diversity, Food consumption, Australia

## Abstract

**Purpose:**

This cross-sectional study aimed to assess the association between ultra-processed foods consumption and dietary diversity and micronutrient intake in Australia.

**Methods:**

As part of the Nutrition and Physical Activity Survey (2011–2012), 12,153 participants aged 2 years and above were recruited and interviewed. Dietary intake data were collected by two 24-h dietary recalls using the Automated Multiple-Pass Method. The NOVA classification system was used to group the food items based on the extent and purpose of industrial food processing. The mean micronutrient contents were calculated for the total diet, and for two diet fractions; one made up entirely of ultra-processed foods (NOVA group 4) and the other consisting of all non-ultra-processed foods (aggregation of NOVA food groups 1 to 3). The mean micronutrient content in the ultra-processed and non-ultra-processed food diet fractions were compared. Dietary diversity was measured using the ten Food Group Indicators (FGI) of the Food and Agriculture Organization and was defined as the sum number of FGIs per individual. Multiple linear regression models were used to assess the association between the quintiles of energy contribution of ultra-processed foods, dietary diversity, and micronutrient intake.

**Results:**

A negative association was found between quintiles of energy contribution of ultra-processed foods and dietary diversity (*β* = − 0.43; *p* < 0.001). The overall micronutrient content was lower in the diet fraction dominated by ultra-processed foods compared to the non-ultra-processed food diet fraction in the study population. The dietary contents of vitamins A, E, C, B9, B12, zinc, calcium, iron, magnesium, potassium, and phosphorus were reduced significantly with increased consumption of ultra-processed foods, even after adjustment for sociodemographic factors and dietary diversity.

**Conclusion:**

The quintiles of energy contribution of ultra-processed foods were negatively associated with dietary diversity and micronutrient intake in Australia.

**Supplementary Information:**

The online version contains supplementary material available at 10.1007/s00394-023-03245-2.

## Introduction

Increasing evidence supports the detrimental impacts of food ultra-processing on human health [1, 2]. Ultra-processed foods (UPFs) are made of processed formulations of low-cost ingredients manufactured with ‘cosmetic’ additives [3, 4]. Nationally representative data have shown that UPFs are contributing to more than half of the dietary energy in some high-income countries [5–7] and about one-third to one-fifth of the energy intake in middle-income nations [8–10]. In Australia [11], France [12], and Japan [13], 30–45% of the daily energy consumption comes from UPFs. Ultra-processed products are becoming dominant in global food systems [14], and Australia alone has experienced a growth rate of 5% in UPFs expenditures between 1989 and 2010 [15].

UPF-rich diets are associated with a wide array of health complications, partly due to low dietary diversification and micronutrient intake. In terms of micronutrients; studies in the US [16], UK [7], Australia [11], Canada [5], Brazil [8], Mexico [17], Chile [18], and Colombia [19] consistently reported that UPF-rich diets are nutritionally unbalanced. In these studies, rises in the share of UPFs were inversely associated with the intake of vitamins A, C, D, E, B12, B6, and β-carotene, thiamine, riboflavin, niacin, folate, zinc, potassium, phosphorus, magnesium, calcium, and iron. In studies in Australia, higher consumption of UPFs was positively associated with non-recommended intake of free sugar, sodium, and saturated fat [11] and negatively related to the overall diet quality [20]. Dietary diversity is known as an important construct of dietary metrics linked to Non-communicable Diseases (NCDs) prevention [21]. In studies among adult men in India [22], primary schoolchildren in Côte d’Ivoire [23], and community-dwelling older people in Thailand [24], low dietary diversity was associated with a higher prevalence of NCDs. In addition to dietary diversity and micronutrient intake, the variety of hyper-palatable foods in UPF-rich diets may promote compulsive eating, which along with the large portion sizes of UPFs, can lead to a excessive energy intake [25]. Also, as a rule, energy-dense UPFs ameliorate the sense of satiety [4], trigger hyperglycemic responses [4], and increase the cardiometabolic and NCDs risk factors [1, 2, 13]. It is estimated that halving UPFs consumption in the UK could reduce cardiovascular disease mortality by 10% by 2030 [26]. Beyond the nutritional facets, industrial food processing can degrade the general characteristics of the original food matrix, which can lead to different health complications [27].

More than half of men and 73% of women aged 2 years and over have a low dietary intake of calcium and 23% of women have different forms of iron deficiency in Australia [28]. Likewise, 7% and 16% of men and women have inadequate intakes of thiamine, respectively, and 9% of women aged 19 years and over have failed to meet their folate requirements from food sources [28]. Given the significance of dietary diversity and micronutrient adequacy in health maintenance across the lifecycle and the connection between inadequate micronutrients intake and higher risk of NCDs, this study aimed to evaluate UPFs consumption in association with dietary diversity and micronutrient intake in Australia. The investigated micronutrients were selected based on data availability and consistent with the literature. Also, UPFs were defined according to the NOVA food classification system. To the best of our knowledge, this is the first study to investigate the NOVA-classified UPFs, dietary diversity, and micronutrient intake in Australia. Australia's unique geographical location relative to other countries can potentially influence the accessibility and availability of specific food items and the food culture, which may limit the generalizability of findings from other countries. Therefore, it is important to study this topic within the Australian context.

## Methods

### Data source

This cross-sectional study is based on the 2011–2012 National Nutrition and Physical Activity Survey (NNPAS) data, part of the 2011–2013 Australian Health Survey (AHS). The NNPAS data collection was conducted between May 2011 and June 2012 on 9,519 households and included a random sample of Australians selected via stratified, multistage probability cluster sampling. As part of the data collection, 12,153 Australians aged 2 years and above were interviewed [29]. This study is reported according to the Strengthening the Reporting of Observational Studies in Epidemiology—Nutritional Epidemiology (STROBE-nut) reporting guidelines (Supplementary Table 1).

Sociodemographic data were collected for all individuals via face-to-face interviews and included age, sex, educational attainment, income, socioeconomic status, and geographical location [30]. As part of the NNPAS, the dietary intake data were collected through two 24-h dietary recalls administered by trained and experienced interviewers using the Automated Multiple-Pass Method. The Automated Multiple-Pass Method involves five steps to assist the interviewers maximize data collection on the amount, timing, cooking method, and processing level of the consumed food items [31]. The first dietary recall was completed in a face-to-face interview (*n* = 12,153) and the second recall was done via a telephone interview (*n* = 7735) conducted eight days or more after the first interview [29]. Dietary information for children aged 2 to 5 years was reported by the child’s parent/guardian (child’s proxy). This method was previously found to be a valid instrument to assess energy intake among children aged 4 to 10 years old [32]. For ages 6–8 years, the child was allowed to assist the proxy and from 9 to 11 years, the child was eligible to be interviewed with the assistance of the proxy, if required [33]. Where permission was granted by a parent/guardian, adolescents aged 12–17 years old were interviewed in person [33], otherwise, questions were answered by the parent/guardian. Energy and micronutrient (vitamins A, C, E, B12, thiamine, riboflavin, niacin, pyridoxine, folate, and zinc, calcium, iron, magnesium, potassium, and phosphorus) intakes were estimated based on the Australian Food Composition Database (AUSNUT 2011–2013). The AUSNUT 2011–2013 Food Composition Database contains information for approximately 5740 foods and beverages and was specifically designed to match the NNPAS dietary intake survey [33, 34]. Given that Australia has certain mandatory food fortification policies, such as flour fortification with folic acid, the nutrition composition of the ingredients and food items takes the micronutrient fortifications into account [35]. We did not analyse information on dietary supplementation.

### NOVA classification

Food and beverages recorded in the NNPAS were previously classified according to the NOVA classification system [11] into the following four groups (and subgroups within these groups): Group 1—Unprocessed or minimally processed foods (e.g. rice and other cereals, meat, fish, milk, eggs, fruit, roots and tubers, vegetables, nuts, and seeds); Group 2—Processed culinary ingredients (e.g. table sugar, plant oils, and butter); Group 3—Processed foods (e.g. processed bread and cheese, canned fruit and fish, and salted and smoked meats); Group 4—Ultra-processed foods (UPFs; e.g. confectionaries, savoury snacks, fast food dishes, mass-produced packaged bread, frozen and ready meals, and soft drinks).

Ultra-processed products, which are of interest in this study, are formulations of low-cost ingredients, many of non-culinary use, that result from a sequence of industrial processes [4]. The manufacture of UPFs starts with the extraction of substances existing in intact foods, such as oils, fats, sugars, starches, and protein [3]. Intermediate processes may involve hydrolysis, hydrogenation, and other chemical modifications of the extracted substances [4]. Other steps include the assembling of modified (e.g., hydrogenated oils) and unmodified (e.g., sugar) substances using procedures such as extrusion and pre-frying, the addition of ‘cosmetic’ additives such as flavours, colours, thickeners, or emulsifiers, and sophisticated packaging with the frequent employment of novel synthetic materials [4]. The presence or absence of these ingredients was identified in accordance with the auxiliary AUSNUT data sources (Food details and Food recipe files), which are based on the list of ingredients on the food packages or the company websites [11]. Food items in this study were classified by two evaluators with expertise in the Australian food supply, the AUSNUT 2011–2013 Food Composition Database, and the NOVA classification system. More information regarding the UPFs’ classification system in Australia can be found elsewhere [11].

### Dietary diversity

Food items were classified according to the ten Food Group Indicators (FGIs) proposed by the Food and Agriculture Organization of the United Nations (FAO) to measure dietary diversity: (1) grain, white roots and tubers, and plantains (starchy staples); (2) pulses (beans, peas, and lentils); (3) nuts and seeds; (4) dairy; (5) meat, poultry, and fish; (6) eggs; (7) dark green leafy vegetables; (8) vitamin A-rich fruits and vegetables; (9) other vegetables; (10) and other fruits [36]. Ultra-processed products were not included in the FGIs based on the premise that these foods are unhealthy and should not be recommended as part of the diversity indicators. Dietary diversity was established as the number of FGIs (>15 g) consumed by each individual per day. Beverages were considered as part of the FGIs. For instance, FGI 4 includes dairy products such as milk, and groups 7–10 describe different types of fruits, which also includes fruit juices. Ultra-processed and unhealthy beverages such as soda and sugary drinks were not included based on the health-deteriorating notion.

### Data analysis

Dietary intake data were adjusted for the Multiple Source Method to account for intra-person variability [37]. The study population excluded women during pregnancy or breastfeeding. The mean contribution (%) of each NOVA food group and subgroup to the total energy intake was calculated. The study population was then stratified into quintiles of the energy contribution of UPFs (first and fifth quintiles representing the lowest and highest consumption of UPFs, respectively). The % energy share of each NOVA food group and subgroup was estimated across those quintiles.

The mean contents of the selected micronutrients were calculated for the total diet (micronutrient density; mg per 1000 kcal) and for two diet fractions made up entirely of ultra-processed (NOVA group 4) versus all non-ultra-processed foods (aggregation of unprocessed or minimally processed foods, processed culinary ingredients and processed foods; NOVA food groups 1 to 3). Independent Samples *t* test was used to assess the mean differences between the two dietary fractions.

The associations between the energy contribution of UPFs with FGIs by socio-demographic characteristics were studied using adjusted Poisson regression models. The mean micronutrient intake and dietary diversity across the quintiles of the energy contribution of UPFs were studied using linear regression models. The first model was adjusted for participants’ age, sex, educational attainment, income, socioeconomic status, and geographical location, and model 2 was additionally adjusted for dietary diversity. The analyses were based on the first 24-h recall data, which is deemed suitable for the estimation of the group means. Finally, sensitivity analyses were conducted: (i) using the exposure (the percentage of energy explained by UPFs) as a continuous variable (Supplementary Table 2); (ii) using energy-adjusted FGIs (Supplementary Table 3).

## Results

Table 1 illustrates the micronutrient density of the total diet and two diet fractions made up of UPFs (NOVA group 4) and non-UPFs (aggregated NOVA groups 1–3). Compared to the UPFs-dominated diet fraction, the fraction made up of non-UPFs had a higher density of all micronutrients (except Vitamins B1, B2, and iron), with differences ranging from 3 times (Vitamin B12) to 1.1 times (Vitamin E) (*p* for all < 0.001). In particular, vitamins B12 and C were about 3 times higher density in the non-UPFs diet fraction compared to the UPFs fraction (*p* < 0.001).

**Table 1:** Dietary diversity and micronutrient content (standardised for 1000 kcal) of the overall diet and the ultra-processed and non-ultra-processed foods diet fractions; Australian population aged 2 + years (NNPAS 2011–2012; *n* = 11,862)

	Overall diet		Ultra-processed food diet fraction		Non-ultra-processed food diet fraction		Ratio non-UPFs/UPFs
	Mean	(SE)	Mean	(SE)	Mean	SE	
*Dietary Diversity*							
Number of Food Group Indicators	5.55	0.02	–	–	5.55	0.02	–
*Vitamins*							
Vitamin A (RAE)	393.8	7.69	204.9	2.23	559	13.5	2.7*
Vitamin B1 Thiamine (mg)	0.85	0.008	1.22	0.02	0.63	0.004	0.5*
Vitamin B2 Riboflavin (mg)	0.93	0.005	1.06	0.01	0.90	0.005	0.9*
Vitamin B3 Niacin (mg)	20.2	0.07	15.8	0.12	24.2	0.11	1.5*
Vitamin B6 Pyridoxine (mg)	0.70	0.005	0.51	0.01	0.85	0.004	1.7*
Vitamin B9 Folate (mg)	142.4	0.70	102.6	1.02	179.3	1.06	1.7*
Vitamin B12 (μg)	2.24	0.02	1.08	0.01	3.21	0.04	3.0*
Vitamin C (mg)	49.2	0.49	44.6	1.20	55.6	0.59	1.2*
Vitamin E (mg)	5.09	0.30	4.84	0.04	5.45	0.04	1.1*
*Minerals*							
Calcium (mg)	394.5	1.91	333.4	3.25	470.4	3.12	1.4*
Iron (mg)	5.68	0.03	6.55	0.07	5.20	0.03	0.8*
Magnesium (mg)	160.8	0.58	130.5	0.84	190.6	0.99	1.5*
Potassium (mg)	1403.7	4.16	1043.3	6.02	1722.8	6.80	1.6*
Phosphorus (mg)	714.8	1.93	590.7	3.82	835.6	2.48	1.4*
Zinc (mg)	5.24	0.02	3.95	0.02	6.31	0.03	1.6*

*NNPAS* National Nutrition and Physical Activity Survey, *SE* Standard Error, *RAE* Retinol Activity Equivalents

*p value for differences with non-ultra-processed foods by using Student’s tests in each micronutrient; *p* < 0.001 is considered significant

^a^Includes NOVA unprocessed or minimally processed foods, processed culinary ingredients and processed foods

Table 2 represents the distribution of sociodemographic variables according to the number of FGIs by the quintiles of the energy contribution of UPFs. The mean percentage of the total energy intake from UPFs was 43.69 ± 0.21. Also, the mean quintiles of energy contribution of UPFs ranged from 20.26% in the first quantile to 68.33% in the fifth quantile. Based on the results, the mean dietary diversity was reduced with increasing energy contribution of the UPFs from quantile 1 to 5 across all sociodemographic groups and subgroups (*p* < 0.001). Additional models were run also adjusting for the effect of energy intake among different age groups (Supplementary Table 3), and consistent results were found.

**Table 2:** Dietary diversity across quintiles of the energy contribution of ultra-processed foods by socio-demographic characteristics; Australian population aged 2 + years (NNPAS 2011–2012; n = 11,862)

Socio-demographic characteristics	Total sample (%)	Mean (SE)	Mean (SE) FGIs by quintiles of energy contribution of ultra-processed foods^¥^				
			1	2	3	4	5
All	100	5.55 (0.02)	6.26 (0.04)	6.05 (0.05)	5.86 (0.05)	5.38 (0.05)	4.19 (0.05)***
*Sex*							
Male	50.7	5.42 (0.03)	6.11 (0.06)	5.94 (0.07)	5.75 (0.07)	5.32 (0.07)	4.12 (0.07)***
Female	49.3	5.68 (0.03)	6.41 (0.06)	6.17 (0.06)	5.98 (0.06)	5.45 (0.07)	4.23 (0.08)***
*Age group (years)*							
2 to 4	4.2	5.09 (0.09)	5.90 (0.22)	5.74 (0.18)	5.43 (0.19)	5.07 (0.19)	4.00 (0.17)***
5 to 11	9.1	5.19 (0.07)	6.79 (0.23)	5.63 (0.16)	5.74 (0.16)	5.62 (0.13)	4.35 (0.12)***
12 to 19	10.8	4.95 (0.08)	5.68 (0.22)	5.98 (0.21)	5.80 (0.16)	5.16 (0.14)	4.03 (0.13)***
20 to 59	56.2	5.65 (0.03)	6.29 (0.05)	6.04 (0.06)	5.92 (0.07)	5.40 (0.07)	4.18 (0.08)***
60 +	19.7	5.85 (0.04)	6.29 (0.07)	6.21 (0.09)	5.90 (0.08)	5.43 (0.11)	4.41 (0.15)***
*Years of education*							
Low (≤ 9)	12.5	5.31 (0.06)	5.79 (0.12)	5.82 (0.12)	5.65 (0.11)	5.07 (0.15)	3.87 (0.17)***
Medium (10 to 12 with no graduate degree)	63.6	5.44 (0.03)	6.21 (0.06)	5.97 (0.06)	5.79 (0.06)	5.37 (0.06)	4.15 (0.06)***
High (12 with graduate degree)	23.9	5.99 (0.05)	6.55 (0.07)	6.35 (0.09)	6.17 (0.09)	5.62 (0.10)	4.60 (0.14)***
*SEIFA*							
Quintile 1—greater disadvantage	17.9	5.18 (0.06)	5.74 (0.10)	5.52 (0.13)	5.82 (0.11)	5.31 (0.12)	3.93 (0.12)***
Quintile 2	19.8	4.35 (0.05)	6.02 (0.10)	5.81 (0.11)	5.62 (0.10)	5.38 (0.10)	4.07 (0.11)***
Quintile 3	20.8	5.54 (0.05)	6.39 (0.10)	6.05 (0.10)	5.88 (0.10)	5.30 (0.10)	4.22 (0.11)***
Quintile 4	18.8	5.72 (0.05)	6.58 (0.09)	6.18 (0.09)	5.87 (0.10)	5.40 (0.11)	4.28 (0.15)***
Quintile 5—greater advantage	22.7	5.88 (0.05)	6.42 (0.07)	6.47 (0.09)	6.07 (0.10)	5.51 (0.11)	4.54 (0.13)***
*Household income*							
Quintile 1—lower income	16.4	5.25 (0.05)	5.86 (0.10)	5.78 (0.11)	5.63 (0.11)	5.09 (0.12)	3.97 (0.13)***
Quintile 2	16.0	5.41 (0.06)	6.02 (0.12)	5.84 (0.11)	5.83 (0.10)	5.57 (0.11)	4.14 (0.13)***
Quintile 3	18.5	5.54 (0.05)	6.42 (0.10)	6.13 (0.10)	5.73 (0.10)	5.41 (0.10)	4.24 (0.12)***
Quintile 4	18.7	5.60 (0.05)	6.45 (0.09)	6.09 (0.11)	5.95 (0.11)	5.39 (0.12)	4.28 (0.12)***
Quintile 5—greater income	17.0	6.02 (0.05)	6.54 (0.09)	6.32 (0.12)	6.27 (0.10)	5.56 (0.11)	4.66 (0.15)***
*Geographical location*							
Major cities of Australia	70.7	5.57 (0.03)	6.28 (0.05)	6.04 (0.05)	5.87 (0.06)	5.33 (0.06)	4.19 (0.07)***
Inner regional Australia	19.7	5.50 (0.05)	6.23 (0.11)	6.09 (0.11)	5.85 (0.11)	5.49 (0.10)	4.20 (0.11)***
Other	9.6	5.47 (0.07)	6.14 (0.13)	6.06 (0.12)	5.81 (0.12)	5.50 (0.14)	4.18 (0.15)***

*NNPAS* National Nutrition and Physical Activity Survey, *SEIFA* Socio-Economic Index for Areas

***p < 0.001 for prevalence ratio estimated using Poisson regression models adjusted by all the sociodemographic characteristics in the table (quintile 1 vs. quintile 5)

^¥^Percentage of total energy intake from ultra-processed foods. Mean (43.69 ± 0.21); quintiles mean and range: Q1 = 20.26 (0 to 28.72); Q2 = 34.08 (28.73 to 38.79); Q3 = 43.22 (38.80 to 47.76); Q4 = 52.59 (47.76 to 58.12); Q5 = 68.33 (58.12 to 100)

**Fig. 1:**
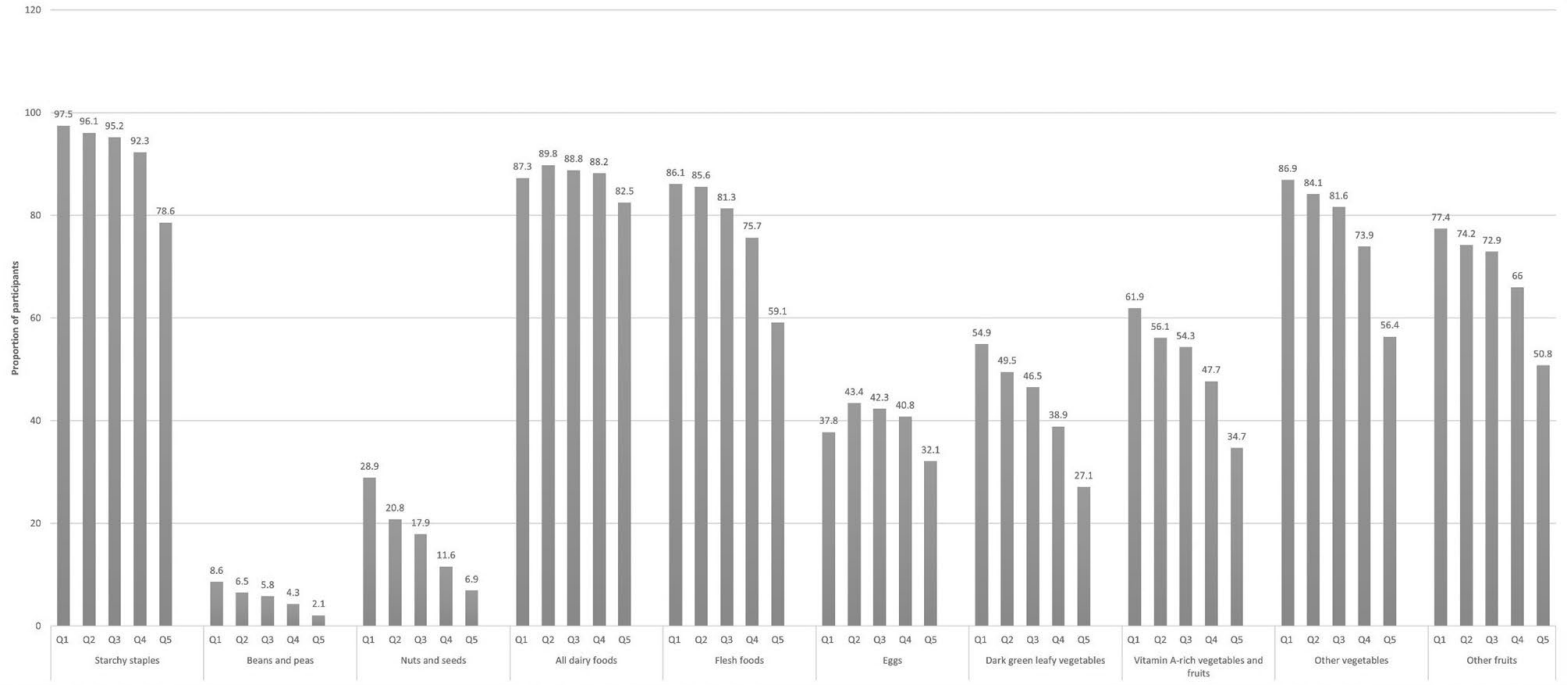
Proportion of participants who consumed each of the 10 Food Group Indicators (FGIs)^†^ across quintiles of energy contribution of ultra-processed foods^¥^. Australian population aged 2+ years (NNPAS 2011–2012) (*n* = 11,862). Notes: *NNPAS* National Nutrition and Physical Activity Survey. ^†^*p* value of linear trend < 0.001 across quintiles using regression models adjusted for age, sex, educational attainment, socio-economic status, income and geographical location was observed for all food groups, except dairy (*p* = 0.09) and eggs (*p* = 0.01). ^¥^Percentage of total energy intake from ultra-processed foods. Mean (43.69 ± 0.21); quintiles mean and range: Q1 = 20.26 (0 to 28.72); Q2 = 34.08 (28.73 to 38.79); Q3 = 43.22 (38.80 to 47.76); Q4 = 52.59 (47.76 to 58.12); Q5 = 68.33 (58.12 to 100)

Figure 1 presents the proportion of the study sample who consumed each of the 10 FGIs across quintiles of the energy contribution of UPFs. As illustrated, there had been linear reductions in the proportion of participants that consumed all the ten FGIs across the UPFs-contributed energy quantiles, except in dairy foods and eggs, where the quintiles indicated inverted U-shapes.

Table 3 represents the distribution of the FGIs and micronutrient intakes across quintiles of the energy contribution of UPFs. After adjustment for potential confounders in model 1, an inverse and statistically significant association was found between dietary diversity and the quintiles of the energy contribution of UPFs (*β* = − 0.43; *p* < 0.001). In the adjusted models, the dietary intakes of vitamins A, E, C, B12, niacin, pyridoxine, folate, zinc, calcium, iron, magnesium, potassium, and phosphorus were negatively associated with the consumption of UPFs (*p* for all < 0.001), while no significant associations at the *p* = 0.001 level were observed for thiamine and riboflavin. Similar results were found when the exposure was used as a continuous variable in the sensitivity analysis (*p* < 0.001; Supplementary Table 2).

**Table 3:** Dietary diversity and micronutrients intake (standardised for 1000 kcal) across the quintiles of the dietary share of ultra-processed foods. Australian population aged 2 + years (NNPAS 2011–2012; n = 11,862)

Nutrients	Quintiles of the dietary contribution of ultra-processed foods (% of total dietary energy)^¥^													
	1		2		3		4		5		Model 1		Model 2	
	Mean	SE	Mean	SE	Mean	SE	Mean	SE	Mean	SE	*β*	SE	*β*	SE
Dietary Diversity														
Number of Food Group Indicators	6.18	0.04	6.01	0.05	5.85	0.05	5.42	0.05	4.31	0.05	− 0.43	0.02***	–	–
*Vitamins*														
Vitamin A (RAE)	458.1	16.6	396.6	9.96	423.4	28.3	373.8	14.5	313.5	8.42	− 30.9	4.40***	− 26.2	4.08***
Vitamin B1 Thiamine(mg)	0.79	0.01	0.87	0.02	0.88	0.02	0.87	0.02	0.85	0.02	0.01	0.006*	0.009	0.006
Vitamin B2 Riboflavin (mg)	0.91	0.01	0.94	0.01	0.93	0.01	0.93	0.01	0.92	0.01	0.0008	0.004	0.0002	0.004
Vitamin B3 Niacin (mg)	21.8	0.17	21.2	0.16	20.3	0.14	19.5	0.14	18.2	0.17	− 0.88	0.06 ***	− 0.85	0.06***
Vitamin B6 Pyridoxine (mg)	0.79	0.01	0.72	0.01	0.68	0.01	0.66	0.01	0.64	0.02	− 0.03	0.004 ***	-0.03	0.004***
Vitamin B9 Folate (mg)	160.8	1.82	148.9	1.52	143.2	1.40	136.7	1.27	122.1	1.48	− 8.93	0.52 ***	− 7.72	0.56***
Vitamin B12 (μg)	2.46	0.04	2.33	0.03	2.30	0.07	2.16	0.04	1.96	0.03	− 0.12	0.01 ***	− 0.12	0.01***
Vitamin C (mg)	56.8	1.23	51.8	1.15	49	0.99	46.1	0.97	41.7	1.22	− 3.59	0.39 ***	− 2.96	0.41***
Vitamin E (mg)	5.86	0.09	5.32	0.06	5.06	0.06	4.81	0.04	4.43	0.05	− 0.33	0.02 ***	− 0.24	0.02***
*Minerals*														
Calcium (mg)	398	4.3	402.6	4.47	398	4.27	393	4.2	373.9	4.46	− 5.64	1.46 ***	− 5.58	1.57***
Iron (mg)	5.87	0.07	5.92	0.06	5.73	0.06	5.67	0.06	5.17	0.07	− 0.16	0.02 ***	− 0.15	0.03***
Magnesium (mg)	183.8	1.5	170.3	1.15	159.9	1.1	152.1	1.09	139.1	1.20	− 10.7	0.44 ***	− 10.1	0.46***
Potassium (mg)	1551.7	10.12	1465	8.69	1408	8	1357	8.25	1237	9.22	− 73.4	3.12 ***	− 64.8	3.30***
Phosphorus (mg)	762.3	4.50	751	4.64	717.5	3.96	697.4	3.73	643.7	4.24	− 28.9	1.46 ***	− 26.2	1.54***
Zinc (mg)	5.84	0.07	5.55	0.05	5.28	0.04	5.05	0.04	4.51	0.04	− 0.32	0.02 ***	− 0.32	0.02***

*NNPAS* National Nutrition and Physical Activity Survey, *RAE* Retinol Activity Equivalents, ^¥^Percentage of total energy intake from ultra-processed foods. Mean (43.69 ± 0.21); quintiles mean and range: Q1 = 20.26 (0 to 28.72); Q2 = 34.08 (28.73 to 38.79); Q3 = 43.22 (38.80 to 47.76); Q4 = 52.59 (47.76 to 58.12); Q5 = 68.33 (58.12 to 100), Model 1: Adjusted for age, sex, educational attainment, socio-economic status, income and geographical location. Model 2: Adjusted for covariates of Model 1 and the 10 FGI

*p < 0.05, **p < 0.01 and ***p < 0.001 for linear trend across quintiles

## Discussion

In this study, dietary diversity and the dietary content of most micronutrients were inversely associated with the energy share of UPFs. Our findings are consistent with other population-based studies in the US [16], Canada [5], Brazil [8], and Mexico [17]. To the best of our knowledge, this is the first study to investigate the consumption of UPFs in association with dietary diversity and micronutrient intake in Australia. In 2019, co-authors of this study used the same data and investigated the dietary intake of macronutrients [11]. The energy contribution of UPFs was positively associated with the dietary intake of total, saturated, and trans fats and negatively linked with dietary fibre intake [11].

As ultra-processed food consumption increases globally, food manufacturers are involving reformulated UPFs in food fortification programs as potential vehicles of micronutrient supplementation regardless of their poor nutritional quality [38]. Although food fortification programs partly address populations’ micronutrient-related malnourishment, fortification of UPFs can maintain or increase their current rates of consumption, thereby resulting in a constant or higher intake of sodium, saturated fats, and sugar and increased risks of NCDs [11]. To tackle the negative consequences of these approaches, it is best to prioritise compliance with the current Australian Dietary Guidelines and the elements of healthy diet, which have a particular emphasis on dietary diversification and consumption of fortified foods that are not ultra-processed [39]. Considering the higher density of micronutrients in unprocessed and minimally processed foods, enhancing dietary diversity is key to achieving the vitamin and mineral intake recommendations at the population level.

In this study, the number of FGIs representing dietary diversity was negatively associated with the energy contribution of UPFs. In addition, the proportion of participants that consumed the ten identified FGIs was higher in quintile 1 of the UPFs energy contribution across all food groups, except for dairy products and eggs. Consistent with the findings of a similar study in Mexico [17], starchy staples were among the highly consumed food group across the UPF quintiles, which decreased from 97.5% in quintile 1 to 78.6% in quintile 5. This can be explained by the fact that corn and rice are still among the primary food sources and essential ingredients of international and Australian cuisines [40].

Compared to minimally processed foods, UPFs may be predominant in the food basket of socioeconomically disadvantaged households in Australia [20, 41]. The significant expense and financial hurdles involved in obtaining nutritious foods pose major barriers to maintaining a healthy diet [41, 42]. This requires exploring the socioeconomic aspects of food processing in the planning and management of public health policies. Recently, Lee and others reported that although recommended healthy diets can cost 20% less than routine diets, they might still be unaffordable for low-income families in Australia [42]. Despite the various interventions and programs in place, the marketing rates of UPFs and the prevalence of obesity and NCDs in Australia have been increasing simultaneously in recent years [11]. This could be a possible sign of a double burden of malnourishment in Australia, which needs to be considered by policymakers when developing dietary guidelines and planning sustainable and equitable food systems.

In this study, the average intake of thiamine, riboflavin, and iron was higher in the UPFs-dominated diet fraction compared with the non-UPFs fraction. Nevertheless, when assessing intakes of thiamine and riboflavin across quintiles of UPF consumption, associations did not remain significant. These results are aligned with similar findings in Brazil [8] and Mexico [17] and can be linked to the high consumption of nutritionally fortified, mass-produced bread and breakfast cereals in the top quintiles of UPFs in Australia [11]. Further investigation is required to evaluate whether these associations remain consistent in recent survey data.

Although extensive food processing is generally criticized, it is important to acknowledge the benefits of processing to societies, as well. This has increased safety and convenience of products, and certain techniques bring benefits such as cooking at normal temperatures, which increases the bioavailability of certain phytonutrients such as lycopene [43]. These are different from the processing techniques applied in UPFs' production, which can alter the food structure and composition and negatively impact the absorption and utilization of nutrients by the body [27].

This study provided evidence to support the detrimental impacts of UPFs on population dietary patterns in Australia. This is consistent with previous studies in different countries and is aligned with the existing dietary guidelines, emphasizing the significance of dietary diversification and prioritization of unprocessed and minimally processed foods over UPFs. Therefore, reducing the share of UPFs in the population's eating patterns can enhance the diet quality in Australia, and help the achievement of micronutrient intake recommendations. Further research in this area is required to validate the findings. Future studies may consider the comparison of population-level micronutrient intake with official dietary guidelines and recommendations in Australia to make firm conclusions.

Our study had several strengths, including the use of the most recent, individual-level dietary data collected from a nationally representative sample of Australian children and adults, which along with the application of valid assessment methods, increases generalizability. Also, in this study, the NOVA food classification system was applied to disaggregated food codes, which enabled the assessment of underestimated food groups and comparisons among different countries. Finally, the assessment of the contribution of foods according to the level of processing to the daily intake of micronutrients provided novel evidence to improve diet quality in Australia. Among the limitations of this study is the collection of dietary data by 24-h recalls, which are subject to errors. A robust method was applied to classify the AUSNUT 2011–2013 food items according to the NOVA system, however, some items may have been misclassified. Also, some items may have been misclassified due to inconsistencies of information indicative of food processing in the datasets. 

## Conclusion

In this nationally representative study of the Australian population, the UPFs contribution to the energy quantiles was negatively associated with dietary diversity and micronutrient. Therefore, promoting dietary diversity, increasing the consumption of unprocessed and minimally processed foods, and discouraging the consumption of UPFs could improve the overall diet quality in Australia.

### Supplementary Information

Below is the link to the electronic supplementary material.

Supplementary file1 (DOCX 82 KB)
